# Message Integration Authentication in the Internet-of-Things via Lattice-Based Batch Signatures

**DOI:** 10.3390/s18114056

**Published:** 2018-11-20

**Authors:** Xiuhua Lu, Wei Yin, Qiaoyan Wen, Kaitai Liang, Liqun Chen, Jiageng Chen

**Affiliations:** 1 State Key Laboratory of Networking and Switching Technology, Beijing University of Posts and Telecommunications, Beijing 100876, China; luxiuhua2011@bupt.edu.cn (X.L.); yinwei24005@bupt.edu.cn (W.Y.); wqy@bupt.edu.cn (Q.W.); 2 Faculty of Mathematics and Information Science, Langfang Normal University, Langfang 065000, China; 3 Department of Computer Science, University of Surrey, Guildford GU2 7XH, UK; k.liang@surrey.ac.uk (K.L.); liqun.chen@surrey.ac.uk (L.C.); 4 School of Computer Science, Central China Normal University, Wuhan 430079, China

**Keywords:** IoT, message integration authentication, batch signature, tree structure, intersection method, lattice

## Abstract

The internet-of-things (also known as IoT) connects a large number of information-sensing devices to the Internet to collect all kinds of information needed in real time. The reliability of the source of a large number of accessed information tests the processing speed of signatures. Batch signature allows a signer to sign a group of messages at one time, and signatures’ verification can be completed individually and independently. Therefore, batch signature is suitable for data integration authentication in IoT. An outstanding advantage of batch signature is that a signer is able to sign as many messages as possible at one time without worrying about the size of signed messages. To reduce complexity yielded by multiple message signing, a binary tree is usually leveraged in the construction of batch signature. However, this structure requires a batch residue, making the size of a batch signature (for a group of messages) even longer than the sum of single signatures. In this paper, we make use of the intersection method from lattice to propose a novel generic method for batch signature. We further combine our method with hash-and-sign paradigm and Fiat–Shamir transformation to propose new batch signature schemes. In our constructions, a batch signature does not need a batch residue, so that the size of the signature is relatively smaller. Our schemes are securely proved to be existential unforgeability against adaptive chosen message attacks under the small integer solution problem, which shows great potential resisting quantum computer attacks.

## 1. Introduction

IoT connects all kinds of objects with the Internet, through various sensing technologies and various means of communication, to achieve remote monitoring and other purposes [1,2,3,4]. Because of large numbers of nodes, wide sources of information and fast updating of information, information authentication processing is very stressful, which brings forward a new research topic for digital signature.

Digital signature was firstly defined and designed in [5,6,7]. This security mechanism allows a message owner to put digital "stamp" on a message to declare the corresponding ownership. Since its introduction, digital signature has been widely employed in many real-world applications, e.g., authentication [8], message integrity check [9], electronic voting, electronic property ownership proof (cryptocurrencies—https://bitcoin.org/en/) and other cloud-based applications [10,11,12]. Due to various of construction techniques, there are many variants of digital signature systems by far, e.g., El-Gamal [13], RSA-based [7], DSA and ECDSA. When it comes to the environment of IoT, batch signature, which is a variant of conventional digital signature, is a good choice.

### 1.1. Batch Signature

The notion of batch signature was firstly proposed by Fiat [14] in CRYPTO 1989. It allows a valid user to sign many messages with almost the cost of one signature operation. In other words, batch signature scheme could sign multiple messages simultaneously. Like carbon paper, someone only needs to sign the top file once by inserting each file in the middle of the carbon paper, all the documents are signed, and each message can be independently verified by recipients. This cryptographic primitive has greatly improved the efficiency of signing a large number of messages.

Following Fiat’s seminal work, a lot of works on batch signature have been proposed. In 1996, M’Raïhi and Naccache [15] gave a batch exponentiation strategy, and applied it to the batch generation of fixed-g-based signatures. In 1999, Pavlovski and Boyd [16] presented a batch signature scheme based on binary tree structure. Binary tree structure is a general construction to transform a common signature algorithm into a batch signature algorithm. In addition, Cheng et al. [17] and Korkmaz [18] analysed the efficiency of existed batch signatures independently.

Besides theoretical construction, there are many more scenarios to apply batch signature technology. In 1999, Boyd et al. [19] proposed an efficient electronic cash using batch signatures. In 2008, Youn et al. [20] applied batch signature in imbalanced communication. We find batch signature is also indispensable in IoT and blockchain. In IoT, when messages from multiple sensor nodes are imported into the host, batch signature of messages is a good way to improve the efficiency of signature. In blockchain, multiple transactions could be handled simultaneously in one-block-generated time. We may save time and space cost by using batch signature scheme.

Faced with a large number of application requirements, the theoretical research of batch signature is not perfect. There are some defects about the existed batch signature, for example, the limited number of signed messages, dependence of signature verification on batch residue and the risk of anti-quantum algorithm attack.

### 1.2. Lattice-Based Signature

The above constructions are based on the traditional number theory assumptions. According to Shor’s results [21], they can not resist the quantum computer’s attack. In the aspect of anti-quantum, lattice-based cryptography is a hot spot for cryptologists, due to the following three advantages. Firstly, large integer factorization and discrete logarithm problems have been proven to be unable to resist quantum computer’s attacks, meanwhile, there is no quantum algorithm that could solve hard problems in lattice. Secondly, cryptographic schemes based on the difficulty assumptions of the average case lattice problems can be reduced to the difficulty assumptions of the worst case lattice problems. It means that the security of cryptographic schemes built on average case lattice problems depends on the worst case lattice problems. The majority of public key cryptosystems are lack of this feature. Thirdly, most of the operations in the lattice are linear operations, so that lattice-based cryptographic schemes have potential computational efficiency.

Lattice-based cryptography has achieved many results. Ajtai [22] proposed the small integer solution problem, known as the SIS problem, in 1996. It is an average case problem hard to solve for appropriate parameter settings, and its difficulty is based on worst case lattice hard problems. The SIS problem, as well as its extension, the inhomogeneous small integer solution problem ISIS, forms the foundation of lattice-based signature schemes.

The most important theoretical breakthrough of lattice-based signature began with the signature scheme in [23]. The main structure of this signature scheme includes a trapdoor generation algorithm and preimage sampleable algorithm; these two algorithms are both with relatively large computational complexity, which hinders the practicability of signature schemes.

In order to solve the efficiency problem of signature schemes, cryptologists have considered the issue from many different perspectives. Alwen and Peikert [24] showed the techniques to get better trapdoor at a faster speed. Micciancio and Peikert [25] proposed a different structure, converted the general lattice trapdoor generation algorithm into a simple lattice trapdoor generation algorithm, and designed a more efficient trapdoor generation algorithm. As a by-product of this new algorithm, the efficiency of preimage sampleable algorithm has also been greatly improved. Therefore, the signature scheme in [25] has better efficiency and security.

Signature schemes in [23,25] have the same construction idea and both belong to the hash-and-sign paradigm. In 2012, Lyubashevsky [26] followed the Fiat–Shamir transformation, managed to avoid the use of trapdoor generation algorithm and preimage sampleable algorithm, and constructed more efficient signature schemes using matrix-vector multiplications and rejecting samplings. These signature schemes make the lattice-based signature schemes practical. Since then, lattice-based signature has continued with more and more contributions, but the core idea still follows the above mentioned signature schemes from [23,25,26].

### 1.3. Our Contributions

In this paper, we propose lattice-based batch signature schemes. Our batch signature schemes remove the batch residue in [19], which makes our batch signature has the same length as one ordinary signature.

1. We propose lattice-based batch signature schemes for the first time. Our schemes possess a general property, that is, our construction can be combined with any existing lattice-based signature scheme.
2. The technique we use is an extension of the intersection method from [27]. The intersection method is as follows: for n—dimensional integer lattices $\Lambda_{1}$ and $\Lambda_{2}$ such that $\Lambda_{1}+\Lambda_{2}=Z^{n}$ and $\Lambda_{1}⋂\Lambda_{2}\neq \phi$, there exists a short vector e, which belongs to $v_{1}+\Lambda_{1}\cap v_{2}+\Lambda_{2}$ and can be viewed as a signature of $v_{1}\in Z^{n}$ and $v_{2}\in Z^{n}$.
  We demonstrate this technique with a concrete example in terms of k≥2. In detail, let $\Lambda_{1}=p_{1}Z^{n}$, $\Lambda_{2}=p_{2}Z^{n},\cdots ,\Lambda_{k}=p_{k}Z^{n}$ with *k* primes $p_{1},p_{2},\cdots ,p_{k}$. Because $p_{1},p_{2},\cdots ,p_{k}$ are different primes, $p_{1}Z^{n}+p_{2}Z^{n}+\cdots +p_{k}Z^{n}=Z^{n}$ and $p_{1}Z^{n}\cap p_{2}Z^{n}\cap \cdots \cap p_{k}Z^{n}=p_{1}p_{2}\cdots p_{k}Z^{n}\neq \phi$. Therefore, for *k* messages $v_{1},v_{2},\cdots ,v_{k}\in Z^{n}$, there exists a short vector $e\in v_{1}+p_{1}Z^{n}\cap v_{2}+p_{2}Z^{n}\cap \cdots \cap v_{k}+p_{k}Z^{n}$, which binds $v_{1},v_{2},\cdots ,v_{k}$ and can be viewed as their batch signature.
3. With the intersection method as core technique, we give two batch signature schemes based on hash-and-sign paradigm and Fiat–Shamir transformation, as well as a lattice-based batch signature scheme based on binary tree.

### 1.4. Organization

Our paper is organized as follows. First, we give some basic definitions and facts about lattice-based cryptography in Section 2. Then, we describe batch signature scheme definition and security in Section 3. In Section 4, we give lattice-based batch signature scheme based on binary tree. In Section 5, we propose lattice-based batch signature scheme based on hash-and-sign paradigm and the intersection method. In Section 6, we demonstrate lattice-based batch signature scheme based on Fiat–Shamir transformation and the intersection method. In Section 7, we present the comparison of our schemes with other lattice-based batch signatures, then describe batch signature’s application to IoT. Finally, we conclude the paper in Section 8.

## 2. Preliminaries

We make use of standard asymptotic notations in our paper. For any function f(n) and g(n) with positive real value set R as range, f=O(g) means that there are constants *a* and *b* such that f(n)≤ag(n) for all n≥b; f=o(g) if and only if $\lim_{n\to \infty }f\left(n\right)/g\left(n\right)=0$; f=ω(g) if and only if g=o(f); f(n) is negligible if and only if f=o(1/g) for any polynomial $g\left(n\right)=n^{c}$; f(n) is overwhelming if and only if 1−f(n) is negligible.

**Definition** **1.** $D_{Z^{m},s,c}$*is the discrete Gaussian distribution in*$Z^{m}$*,its center is*c*and Gaussian parameter is s. If the center is vector*0, 0*may be omitted. If*$e⟵D_{Z^{m},s}$*,its Euclidean norm is*$∥e∥\leq s\sqrt{m}$*with overwhelming probability [23].*

**Definition** **2.** *Trapdoor generation algorithm TrapGen*(n,q,m)*inputs n, q and m, where n is an integer,*q≥3*is an odd, and*m=⌈6nlogq⌉*is the minimum integer not less than*6nlogq*.The algorithm outputs a pair*$\left(A\in Z_{q}^{n\times m},T\in Z^{m\times m}\right)$*such that*A*is statistically close to a uniform random matrix in*$Z_{q}^{n\times m}$, T*is a basis for*$\Lambda_{q}^{⊥}\left(A\right)$*satisfying*$∥\tilde{T}∥\leq O\left(\sqrt{n\log q}\right)$*and*∥T∥≤O(nlogq)*with overwhelming probability. Here,*$\tilde{T}$*is the Gram-Schmidt orthogonalization matrix of*T, ∥T∥*denotes the largest Euclidean norm of the column vectors in matrix*T*[24].*

**Definition** **3.** *Let*$A\in Z_{q}^{n\times m}$*,*T*is a basis for*$\Lambda_{q}^{⊥}\left(A\right)$*,and*$s\geq ∥\tilde{T}∥\cdot \omega \left(\sqrt{\log m}\right)$. *Then for*$u\in Z_{q}^{n}$*,preimage sampleable algorithm SamplePre*(A,T,u,s)*samples*x*satisfying*∥x∥$\leq s\sqrt{m}$*and*Ax=umodq*[23].*

**Definition** **4.** *Small integer solution (SIS) [23]*

   *SIS problem is defined as: for integer q, real β and matrix* $A\in Z_{q}^{n\times m}$*,* *search an integer vector* $e\in Z^{m}$ *satisfying* Ae=0modq*,* ∥e∥≤β *and* e≠0.

**Definition** **5.** *The intersection of lattice* $\Lambda_{1}$ *and lattice* $\Lambda_{2}$ *is not empty, and* $\Lambda_{1}+\Lambda_{2}=Z^{n}$ *for element wise addition.* $v_{1},v_{2}\in Z^{n}$ *are the coset representatives of* $\Lambda_{1}$ *and* $\Lambda_{2}$ *,* *respectively. Then there exists a vector* $e\in Z^{n}$ *such that* $e=v_{1}\;\mathrm{mod}\;\Lambda_{1}$ *and* $e=v_{2}\;\mathrm{mod}\;\Lambda_{2}$ *.* *This result can be generalized to multiple lattices [27].*

**Definition** **6.** *Target Collision Resistant (TCR) Hash function [28]*

   *Let* $h:{\left\{0,1\right\}}^{n}\to {\left\{0,1\right\}}^{m}$ *is a collision-resistant hash function if it satisfies the following properties:*

- *(length-compressing):*m<n
- *(hard to find collisions): For all PPT A, there exists a negligible function ϵ such that for all security parameters*n∈N,
  $$Pr\left[\left(x_{0},x_{1}\right)\leftarrow A\left(1^{n},h\right):x_{0}\neq x_{1}\cap h\left(x_{0}\right)=h\left(x_{1}\right)\right]⩽\epsilon \left(n\right)$$

## 3. System Definition and Threat Model

In this section, we give batch signature definitions of generic algorithms and security, which divide into two parts.

### 3.1. Definition of Batch Signature System

Batch signature can use a signing action to complete the signing of a number of different messages, and the verification of individual message is independent. Besides, the system setup algorithm and key generation algorithm in batch signature scheme are as same as that of ordinary signature scheme.

- **Setup**(λ): Inputting security parameter λ, this algorithm determines necessary system public parameters PP.
- **KeyGen**(λ): With security parameter λ and system parameters PP as above, this algorithm provides public verification key vk and secret signing key sk.
- **Sign**($sk,\{\varpi_{1},\cdots ,\varpi_{k}\}$): Given signing key sk and messages set $\left\{\varpi_{1},\cdots ,\varpi_{k}\right\}$, this algorithm computes batch signature *e*.
- **Verify**($vk,\varpi_{j},e,j=1,\cdots ,k$): Given message $\varpi_{j}$ and its signature *e* associated with verification key vk, this algorithm tells whether the *j*-th message has gained valid authentication, and outputs 1 if the answer is yes, otherwise outputs 0.

### 3.2. Threat Model

Batch signature scheme should also satisfy existential unforgeability against adaptive chosen message attacks (EUF-CMA). We introduce a challenger C and an adversary A interacting with each other in the next game, to describe batch signature scheme’s security.

- **Initialization**: In this period, challenger C executes algorithms **Setup** and **KeyGen**, provides system public parameters PP and public verification key vk for adversary A.
- **Signing queries**: In this stage, adversary A selects a set of messages $\left(\varpi_{1},\varpi_{2},\cdots ,\varpi_{k}\right)$, sends the messages’ set to challenger C for the associated signature. Challenger C invokes **Sign** algorithm, returns the result to adversary A. Adversary A may repeat the query polynomial times in his favorite manner.
- **Forgery**: Once adversary A terminates signing queries, he offers a new message-signature pair $\left(\varpi_{1}^{*},\varpi_{2}^{*},\cdots ,\varpi_{k}^{*},e\right)$.

If message-signature pair $\left(\varpi_{1}^{*},\varpi_{2}^{*},\cdots ,\varpi_{k}^{*},e\right)$ is valid and has not been queried, adversary A wins the game.

**Theorem** **1.** *Batch signature scheme is existential unforgeability against adaptive chosen message attacks(EUF-CMA), if for all adversary A with polynomial bounded computational power, the probability of he wins above game is negligible.*

## 4. Lattice-Based Batch Signature with Binary Tree

### 4.1. Proposed Construction

**Figure 1:**
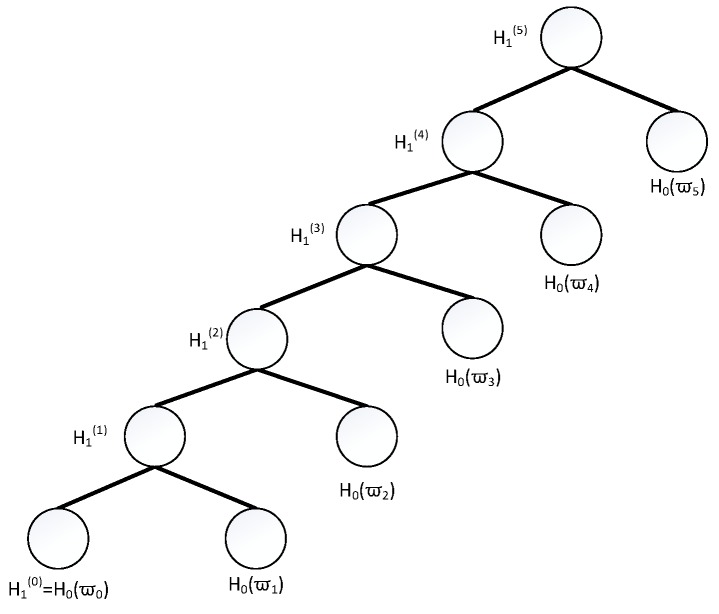
The Schematic of the Binary Tree.

In this part, we combine the signature scheme in [23] and the structure of binary tree in [19], propose the first lattice-based batch signature scheme. The scheme includes the following steps, and the Figure 1 shows the binary tree for message processing.

- **Setup**(λ): In this stage, system parameters are provided with knowledge of security parameter λ.
  1. *n* is a polynomial of λ, q≥3 is a polynomial of *n*, m=⌈6nlogq⌉, $t=O(\sqrt{n\log q})$.
  2. *k* is the number of messages to batch sign, and $s\geq t\cdot \omega (\sqrt{\log m})$ is the Gaussian parameter.
  3. $H_{0}:{\left\{0,1\right\}}^{*}⟶Z_{q}^{n}$ and $H_{1}:Z_{q}^{2n}⟶Z_{q}^{n}$ are two collision resistant hash functions.
- **KeyGen**(λ): With system parameters as above, public verification key vk and secret signing key sk are obtained as follows. Invoke trapdoor generation algorithm TrapGen(n,q,m) to get a uniform and random matrix $A\in Z_{q}^{n\times m}$, and the short basis $T\in Z^{m\times m}$ for lattice $\Lambda_{q}^{⊥}\left(A\right)$ with $∥\tilde{T}∥\leq t$.
  Finally output vk=A, sk=T.
- **Sign**($sk,\left\{\varpi_{0},\cdots ,\varpi_{k-1}\right\}\in {\left\{{\left\{0,1\right\}}^{*}\right\}}^{k}$): Given sk=T and the set of messages $\left\{\varpi_{0},\cdots ,\varpi_{k-1}\right\}\in {\left\{{\left\{0,1\right\}}^{*}\right\}}^{k}$, the following steps lead to batch signature on such messages.
  1. Compute $H_{0}\left(\varpi_{0}\right)$, $H_{0}\left(\varpi_{1}\right)$,⋯, $H_{0}\left(\varpi_{k-1}\right)$, let $H_{1}^{\left(0\right)}=H_{0}\left(\varpi_{0}\right)$, and execute for-loop as follows.
    for i=1 to k−1:
      $H_{1}^{\left(i\right)}=H_{1}\left(H_{1}^{\left(i-1\right)}∥H_{0}\left(\varpi_{i}\right)\right)$
      i=i+1
  2. Compute e= SamplePre$\left(A,T,H_{1}^{\left(k-1\right)},s\right)$.
  3. For $\varpi_{i},i=0,\cdots ,k-1$, compute its brother $B_{i}$. Firstly, $B_{0}=\left(H_{0}\left(\varpi_{1}\right),R\right)$, the left are shown in the next for-loop.
    for i=1 to k−1:
      $B_{i}=\left(H_{1}^{\left(i-1\right)},L\right)$
      i=i+1
    Here, for $\varpi_{i}$’s brother $B_{i}$, its first entry denotes $\varpi_{i}$’s brother note, its second entry denotes the brother locates on $\varpi_{i}$’s left (L) or right (R).
  4. For $\varpi_{i},i=0,\cdots ,k-1$, compute its residue $R_{i}$. At first,
    $\begin{array}{l} R_{0}=\left\{\left(H_{0}\left(\varpi_{1}\right),R\right),\left(H_{0}\left(\varpi_{2}\right),R\right),\cdots ,\left(H_{0}\left(\varpi_{k-1}\right),R\right)\right\}\\ \;\;=\{B_{0},\left(H_{0}\left(\varpi_{2}\right),R\right),\cdots ,\left(H_{0}\left(\varpi_{k-1}\right),R\right)\} \end{array}$,
    the left are shown in the next for-loop.
    for i=1 to k−2:
      $\begin{array}{l} R_{i}=\left\{\left(H_{1}^{\left(i-1\right)},L\right),\left(H_{0}\left(\varpi_{i+1}\right),R\right),\cdots ,\left(H_{0}\left(\varpi_{k-1}\right),R\right)\right\}\\ \;=\{B_{i},\left(H_{0}\left(\varpi_{i+1},R\right),\cdots ,\left(H_{0}\left(\varpi_{k-1}\right),R\right)\right\} \end{array}$
      i=i+1
    When i=k−1, $R_{i}=B_{k-1}=\left\{\left(H_{1}^{\left(k-2\right)},L\right)\right\}$.
    Here, $\varpi_{i}$’s residue $R_{i}$ includes $\varpi_{i}$’s brother $B_{i}$ and all of its ancestor nodes’s brothers.
  5. For $\varpi_{i},i=0,\cdots ,k-1$, its signature is $\left(e,R_{i}\right)$.
- **Verify**($vk,\varpi_{j},\left(e,R_{j}\right),j=0,\cdots ,k-1$): Given message $\varpi_{j}$ and its signature $\left(e,R_{j}\right)$ associated with verification key vk=A,
  1. $H_{1}^{\left(k-1\right)}$ should be recovered firstly.
    (1) If j=0,
    $\begin{array}{l} R_{0}=\left\{\left(H_{0}\left(\varpi_{1}\right),R\right),\left(H_{0}\left(\varpi_{2}\right),R\right),\cdots ,\left(H_{0}\left(\varpi_{k-1}\right),R\right)\right\}\\ \;\;\;\;\;=\left\{B_{0},\left(H_{0}\left(\varpi_{2}\right),R\right),\cdots ,\left(H_{0}\left(\varpi_{k-1}\right),R\right)\right\} \end{array}$,
    for i=1 to k−1:
      $H_{1}^{\left(i\right)}=H_{1}\left(H_{1}^{\left(i-1\right)}∥H_{0}\left(\varpi_{i}\right)\right)$
      i=i+1
    When for-loop terminates, $H_{1}^{\left(k-1\right)}$ is obtained.
    (2) If j≠0,
    $\begin{array}{l} R_{j}=\left\{\left(H_{1}^{\left(j-1\right)},L\right),\left(H_{0}\left(\varpi_{j+1}\right),R\right),\cdots ,\left(H_{0}\left(\varpi_{k-1}\right),R\right)\right\}\\ \;\;\;\;=\left\{B_{j},\left(H_{0}\left(\varpi_{j+1}\right),R\right),\cdots ,\left(H_{0}\left(\varpi_{k-1}\right),R\right)\right\} \end{array}$,
    for i=j to k−1:
      $H_{1}^{\left(i\right)}=H_{1}\left(H_{1}^{\left(i-1\right)}∥H_{0}\left(\varpi_{i}\right)\right)$
      i=i+1
    When for-loop terminates, $H_{1}^{\left(k-1\right)}$ is obtained.
  2. Check whether $∥e∥\leq s\sqrt{m}$ and $Ae=H_{1}^{\left(k-1\right)}\;\mathrm{mod}\;q$. If both relations are true, return 1 and accept the message-signature pair $\left(\varpi_{j},\left(e,R_{j}\right)\right)$; otherwise, return 0 and reject the message-signature pair.

### 4.2. Security Analysis

Correctness of the scheme comes from the preimage sampleable algorithm. According to Definition 3, for messages set $\left\{\varpi_{1},\varpi_{2},\cdots ,\varpi_{k}\right\}$, assume the root of the binary tree is $H_{1}^{\left(k-1\right)}$, due to e= SamplePre$\left(A,T,H_{1}^{\left(k-1\right)},s\right)$, $∥e∥\leq s\sqrt{m}$ and $Ae=H_{1}^{\left(k-1\right)}\;\mathrm{mod}\;q$ hold. Moreover, without secret signing key T, no one can call preimage sampleable algorithm to get a vector e that meets the verification criteria. Therefore, there is no problem with the correctness of the scheme.

Security of the scheme comes from the following Theorem 2.

**Theorem** **2.** *If SIS problem is hard to solve, the lattice-based batch signature scheme based on binary tree has existential unforgeability against adaptive chosen message attacks (EUF-CMA).*

**Proof.**  We assume that adversary A has breached the signature scheme, taking advantage of this attack power, challenger C can solve SIS problem for matrix $A\in Z_{q}^{n\times m}$. Because SIS problem is a hard problem, we ca not find the answer to SIS instance A, which conflicts with our result. In this way, we get that no such adversary exists, and our scheme is secure.

- **Initialization**: In this period, challenger C executes setup algorithm to set system parameters, he sets public verification key vk=A, sends all of them to adversary A.
- **Hash queries**: Challenger C creates a list to save the binary tree for *k* messages, and sets $H=\{(\left(\varpi_{0},\cdots ,\varpi_{k-1}\right),\left(H_{0}\left(\varpi_{0}\right),\cdots ,H_{0}\left(\varpi_{k-1}\right)\right),$ $\left(H_{1}^{\left(1\right)},\cdots ,H_{1}^{\left(k-1\right)}),e)\right\}$.
  When adversary A sends a set of messages $\left(\varpi_{0},\cdots ,\varpi_{k-1}\right)$ to challenger C for hash values. C searches list H,
  If $\left(\varpi_{0},\cdots ,\varpi_{k-1}\right)$ do not exist in list H, C chooses $e⟵D_{Z^{m},s}$, sets $H_{1}^{\left(k-1\right)}=\mathrm{Ae}\;\mathrm{mod}\;q$. Then C randomly picks $H_{0}\left(\varpi_{i}\right)$, i=0,⋯,k−1, sets $H_{1}^{\left(i\right)}=H_{1}\left(H_{1}^{\left(i-1\right)}∥H_{0}\left(\varpi_{i}\right)\right)$ for i=1 to k−1, here $H_{1}^{\left(0\right)}=H_{0}\left(\varpi_{0}\right)$. C saves $(\left(\varpi_{0},\cdots ,\varpi_{k-1}\right),$ $(H_{0}\left(\varpi_{0}\right),\cdots ,H_{0}\left(\varpi_{k-1}\right)),$ $\left(H_{1}^{\left(1\right)},\cdots ,H_{1}^{\left(k-1\right)}),e\right)$ in the list H.
  If $\left(\varpi_{0},\cdots ,\varpi_{k-1}\right)$ exist in list H, C does nothing.
  At last, C returns $(\left(H_{0}\left(\varpi_{0}\right),\cdots ,H_{0}\left(\varpi_{k-1}\right)\right),$ $\left(H_{1}^{\left(1\right)},\cdots ,H_{1}^{\left(k-1\right)})\right)$ to adversary A.
- **Signature queries**: In this stage, adversary A selects a set of messages $\left(\varpi_{0},\cdots ,\varpi_{k-1}\right)$, sends the messages’ set to challenger C for the associated signature. Challenger C firstly searches list H for the messages’ set. If it exists, C returns $\left(\left(H_{0}\left(\varpi_{0}\right),\cdots ,H_{0}\left(\varpi_{k-1}\right)\right),\left(H_{1}^{\left(1\right)},\cdots ,H_{1}^{\left(k-1\right)}\right),e\right)$ to adversary A. If the messages’ set does not exist, C executes hash query firstly. Adversary A may repeat the query polynomial times in his favorite manner.
- **Forgery**: Once adversary A terminates signing queries, he forges a valid message-signature pair $(\left(\varpi_{0}^{*},\cdots ,\varpi_{k-1}^{*}\right),\left(H_{0}\left(\varpi_{0}^{*}\right),\cdots ,H_{0}\left(\varpi_{k-1}^{*}\right)\right),(H_{1*}^{\left(1\right)},\cdots ,$$H_{1*}^{\left(k-1\right)}),e^{*})$.

C searches $(\left(\varpi_{0}^{*},\cdots ,\varpi_{k-1}^{*}\right),\left(H_{0}\left(\varpi_{0}^{*}\right),\cdots ,H_{0}\left(\varpi_{k-1}^{*}\right)\right),$ $\left(H_{1*}^{\left(1\right)},\cdots ,H_{1*}^{\left(k-1\right)}\right),e^{\prime })$ in list H, then computes $e^{\prime }-e^{*}$ as the solution to the SIS instance A, and the analysis is as following.

Due to the validity of the message-signature pair, adversary A has not made signing query on $\left(\varpi_{0}^{*},\cdots ,\varpi_{k-1}^{*}\right)$, and hash query on $\left(\varpi_{0}^{*},\cdots ,\varpi_{k-1}^{*}\right)$ has been done. Given $H_{1*}^{\left(k-1\right)}$, according to preimage min-entropy property of hash function [23], the min-entropy of $e^{*}$ is ω(logn), so that $e^{\prime }-e^{*}\neq 0$ with overwhelming probability. Because $e^{\prime }⟵D_{Z^{m},s}$, $∥e^{\prime }∥\leq s\sqrt{m}$. $∥e^{*}∥\leq s\sqrt{m}$ depends on the validity of forged signature. Therefore, $∥e^{\prime }-e^{*}∥\leq 2s\sqrt{m}$.   □

## 5. Lattice-Based Batch Signature Based on Hash-and-Sign Paradigm

**Figure 2:**
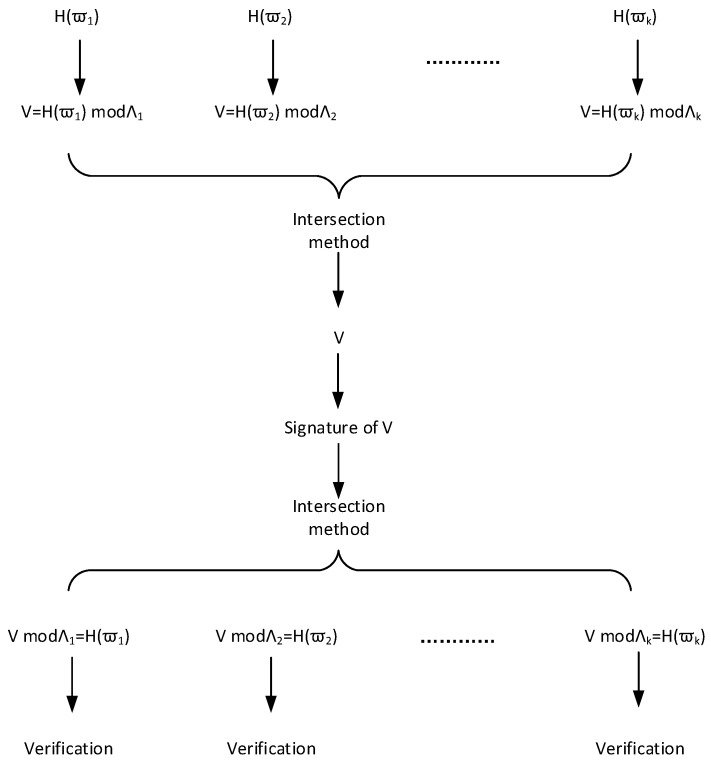
The Schematic of the Intersection Method.

Lattice-based batch signature scheme based on binary tree is successfully constructed and proved, but the signature should associate with all other messages in the batch to complete signature verification, and batch signature length is, thus, longer. Inspired by [27,29], we make use of an intersection method to accomplish the second and third lattice-based batch signature schemes. These two schemes’ signature verification does not require involvement of other messages, so that the length of the signature is shorter, and their schematic of algorithms is shown in the Figure 2.

### 5.1. Design

Here is our second lattice-based batch signature scheme, which follows the hash-and-sign paradigm and the core technique is the intersection method.

- **Setup**(λ): In this stage, system parameters are provided with knowledge of security parameter λ.
  1. *n* is a polynomial of λ, q≥3 is a polynomial of *n*, m=⌈6nlogq⌉, $l=O(\sqrt{n\log q})$.
  2. *k* is the number of messages to batch sign, and $s\geq l\cdot \omega (\sqrt{\log m})$ is the Gaussian parameter.
  3. $H:{\left\{0,1\right\}}^{*}⟶Z^{n}$ is a collision resistant hash function.
- **KeyGen**(λ): With system parameters as above, public verification key vk and secret signing key sk are obtained in the following manners.
  1. Invoke trapdoor generation algorithm TrapGen(n,q,m) to get a uniform and random matrix $A\in Z_{q}^{n\times m}$, and the short basis $T\in Z^{m\times m}$ for lattice $\Lambda_{q}^{⊥}\left(A\right)$ with $∥\tilde{T}∥\leq l$.
  2. Compute *k* different lattices $\Lambda_{i},i=1,\cdots ,k$, such that $\Lambda_{1}+\Lambda_{2}+\cdots +\Lambda_{k}=Z^{n}$ and $\Lambda_{1}\cap \Lambda_{2}\cap \cdots \cap \Lambda_{k}=\Lambda$, which takes *q* as modulus.
    Then $vk=(A,\Lambda ,\Lambda_{i},i=1,\cdots ,k)$, sk=T.
- **Sign**($sk,\left\{\varpi_{1},\cdots ,\varpi_{k}\right\}\in {\left\{{\left\{0,1\right\}}^{*}\right\}}^{k}$): Given sk=T and the set of messages $\left\{\varpi_{1},\cdots ,\varpi_{k}\right\}\in {\left\{{\left\{0,1\right\}}^{*}\right\}}^{k}$, the following steps lead to batch signature on such messages.
  1. Construct equations:
    $$\left\{\begin{array}{c} v=H\left(\varpi_{1}\right)\mathrm{mod}\Lambda_{1}\\ v=H\left(\varpi_{2}\right)\mathrm{mod}\Lambda_{2}\\ \cdots \\ v=H\left(\varpi_{k}\right)\mathrm{mod}\Lambda_{k} \end{array}\right.$$
    compute its solution v.
  2. Invoke preimage sampleable algorithm SamplePre(A,T,v,s) to get the signature e.
- **Verify**($vk,\varpi_{j},e)$: For the *j*-th message $\varpi_{j}$ and the signature e, validation involves the following two relations:
  1. $∥e∥\leq s\sqrt{m}$.
  2. $Ae=H\left(\varpi_{j}\right)\mathrm{mod}\Lambda_{j}$.
    If they are both true, accept message $\varpi_{j}$; otherwise, reject it.

### 5.2. Security Analysis

Correctness of the second scheme is similar to that of the first scheme. By Definition 3, $∥e∥\leq s\sqrt{m}$ and Ae=vmodq. By Definition 2, $v=H\left(\varpi_{j}\right)\;\mathrm{mod}\;\Lambda_{j}$. Combining Ae=vmodq and $v=H\left(\varpi_{j}\right)\;\mathrm{mod}\;\Lambda_{j}$, $\mathrm{Ae}=H\left(\varpi_{j}\right)\;\mathrm{mod}\;\Lambda_{j}$. Moreover, without signing key T, nobody can invoke preimage sampleable algorithm to get a vector e satisfying the verification relations.

Security of the scheme comes from the following Theorem 3.

**Theorem** **3.** *If SIS problem is a hard problem, the lattice-based batch signature scheme based on hash-and-sign paradigm has existential unforgeability against adaptive chosen message attacks (EUF-CMA).*

**Proof.**  If there exists an adversary A who has the ability to forge batch signature for some messages, there exists a challenger C has the ability to give the solution to SIS instance $A\in Z_{q}^{n\times m}$, here the challenger C will seek the help of the adversary A. Since SIS problem is a hard problem, the solution to SIS instance A is hard to obtained, this is in contradiction with our result. Therefore, the adversary A who has the ability to forge batch signature does not exist, and our lattice-based batch signature scheme based on hash-and-sign paradigm has EUF-CMA security.

- **Initialization**: In this period, challenger C sets appropriate system parameters, lets public verification key vk=A, sends all of them to adversary A.
- **Hash queries**: Challenger C creates a list to save the hash values for *k* messages, and sets $H=\{\left(\left(\varpi_{1},\cdots ,\varpi_{k}\right),\left(H\left(\varpi_{1}\right),\cdots ,H\left(\varpi_{k}\right)\right),e\right)\}$.
  When adversary A sends a set of messages $\left(\varpi_{1},\cdots ,\varpi_{k}\right)$ to challenger C for hash values. C searches list H.
  If $\left(\varpi_{1},\cdots ,\varpi_{k}\right)$ exist in list H, C returns $\left(H\left(\varpi_{1}\right),\cdots ,H\left(\varpi_{k}\right)\right)$ directly.
  If $\left(\varpi_{1},\cdots ,\varpi_{k}\right)$ do not exist in list H, C samples $e⟵D_{Z^{m},s}$, sets v=Aemodq, and lets $v\;\mathrm{mod}\;\Lambda_{i}=H\left(\omega_{i}\right)$, i=1,⋯,k. Then C saves $\left(\left(\varpi_{1},\cdots ,\varpi_{k}\right),\left(H\left(\varpi_{1}\right),\cdots ,H\left(\varpi_{k}\right)\right),e\right)$ in list H, and returns $\left(H\left(\varpi_{1}\right),\cdots ,H\left(\varpi_{k}\right)\right)$ to adversary A.
- **Signing queries**: In this stage, adversary A selects a set of messages $\left(\varpi_{1},\varpi_{2},\cdots ,\varpi_{k}\right)$, sends the messages’ set to challenger C for the associated signature. Challenger C searches list H for messages $\left(\varpi_{1},\varpi_{2},\cdots ,\varpi_{k}\right)$.
  If the messages exist in list H, challenger C returns e directly.
  If the messages do not exist in list H, Challenger C firstly executes Hash query, then returns e to adversary A.
- **Forgery**: Once adversary A terminates signing queries, he offers a new message-signature pair $\left(\varpi_{1}^{*},\varpi_{2}^{*},\cdots ,\varpi_{k}^{*},e^{*}\right)$.
  Challenger C searches $(\left(\varpi_{1}^{*},\varpi_{2}^{*},\cdots ,\varpi_{k}^{*}\right),(H\left(\varpi_{1}^{*}\right),$ $\cdots ,H\left(\varpi_{k}^{*}\right)),e^{\prime })$ in list H, then computes $e^{*}-e^{\prime }$ as the solution to the SIS instance A.
  Because message-signature pair $\left(\varpi_{1}^{*},\varpi_{2}^{*},\cdots ,\varpi_{k}^{*},e^{*}\right)$ is valid, adversary A has not made signing query on $\left(\varpi_{1}^{*},\cdots ,\varpi_{k}^{*}\right)$, and hash query on $\left(\varpi_{1}^{*},\cdots ,\varpi_{k}^{*}\right)$ has been done. Given $Ae^{\prime }=v\;\mathrm{mod}\;q$, according to preimage min-entropy property of hash function [23], the min-entropy of $e^{*}$ is ω(logn), so that $e^{\prime }-e^{*}\neq 0$ with overwhelming probability. Because $e^{\prime }⟵D_{Z^{m},s}$, $∥e^{\prime }∥\leq s\sqrt{m}$. $∥e^{*}∥\leq s\sqrt{m}$ depends on the validity of forged signature. Therefore, $∥e^{\prime }-e^{*}∥\leq 2s\sqrt{m}$.

   □

## 6. Lattice-Based Batch Signature Based on FS Transformation

In lattice-based cryptography, trapdoor generation algorithm (Definition 2) and preimage sampleable algorithm (Definition 3) are fundamental algorithms of signature scheme, but both algorithms have high computational complexity. To improve signature scheme’s efficiency, we take lattice signature based on Fiat–Shamir transformation in [26], apply to lattice-based batch signature with intersection method, obtain a new and more efficient batch signature scheme.

### 6.1. Design

- **Setup**(*n*): In this stage, system parameters are provided with knowledge of security parameter *n*.
  1. *q* may be $2^{25}$, *d* may be 1, *r* may be 512.
  2. m>64+n·logq/log(2d+1), κ satisfies $2^{\kappa }\cdot \left(\begin{array}{c} n\\ \kappa \end{array}\right)\geq 2^{100}$.
  3. *s* may be $12d\kappa \sqrt{m}$, and *M* may be $e^{\left(12d\kappa \sqrt{m}/s+{\left(d\kappa \sqrt{m}/\left(2s\right)\right)}^{2}\right)}$.
  4. $H_{1}:{\left\{0,1\right\}}^{*}⟶Z^{n}$ and $H_{2}:{\left\{0,1\right\}}^{*}{⟶\{g:g\in \left\{-1,0,1\right\}}^{r}{,∥g∥}_{1}\leq \kappa \}$ are collision resistant hash functions, where ${∥g∥}_{1}$ is the 1-norm of vector g, namely, it is the sum of the absolute values of each element of vector g.
- **KeyGen**(*n*): With system parameters as above, public verification key vk and secret signing key sk are obtained in the following manners.
  1. Select $S\leftarrow {\left\{-d,\cdots ,0,\cdots ,d\right\}}^{m\times r}$ randomly as secret signing key.
  2. Select $A\leftarrow Z_{q}^{n\times m}$, compute T=AS as public verification key.
  3. Compute *k* different lattices $\Lambda_{i},i=1,\cdots ,k$, such that $\Lambda_{1}+\Lambda_{2}+\cdots +\Lambda_{k}=Z^{n}$ and $\Lambda_{1}\cap \Lambda_{2}\cap \cdots \cap \Lambda_{k}=\Lambda$, which takes *q* as modulus.
    Then $vk=(A,T,\Lambda ,\Lambda_{i},i=1,\cdots ,k)$, sk=S.
- **Sign**($sk,\left\{\varpi_{1},\cdots ,\varpi_{k}\right\}\in {\left\{{\left\{0,1\right\}}^{*}\right\}}^{k}$): Given sk=S and the set of messages $\left\{\varpi_{1},\cdots ,\varpi_{k}\right\}\in {\left\{{\left\{0,1\right\}}^{*}\right\}}^{k}$, the following steps lead to batch signature on such messages.
  1. Construct equations:
    $$\left\{\begin{array}{c} v=H_{1}\left(\varpi_{1}\right)\mathrm{mod}\Lambda_{1}\\ v=H_{1}\left(\varpi_{2}\right)\mathrm{mod}\Lambda_{2}\\ \cdots \\ v=H_{1}\left(\varpi_{k}\right)\mathrm{mod}\Lambda_{k} \end{array}\right.$$
    compute its solution v.
  2. Sample $y\leftarrow D_{Z^{m},s}$ randomly, compute $c=H_{2}\left(\mathrm{Ay},v\right)$.
  3. Let z=Sc+y, output (v,z,c) as signature with probability $\min(\frac{D_{Z^{m},s}\left(z\right)}{MD_{Z^{m},s,\mathrm{Sc}}\left(z\right)},1)$.
- **Verify**($vk,\varpi_{j},\left(v,z,c\right))$: For the *j*-th message $\varpi_{j}$ and the signature (v,z,c), validation involves the following three relations:
  1. $∥z∥\leq 2s\sqrt{m}$.
  2. $c=H_{2}\left(\mathrm{Az}-\mathrm{Tc},v\right)$.
  3. $v\mathrm{mod}\Lambda_{j}=H_{1}\left(\varpi_{j}\right)$.
    If they are true, accept message $\varpi_{j}$; otherwise, reject it.

### 6.2. Security Analysis

The batch signature scheme in Section 5 and the batch signature scheme in Section 6 are different in terms of basic signature schemes: the first basic signature scheme comes from the literature [23], the second basic signature scheme comes from the literature [26]. According to literature [30], for the same security, the second bath signature scheme has better efficiency.

The same as batch signature scheme in Section 5, the batch signature scheme in Section 6’s correctness and security can be reduced to its basic signature scheme, and the details are omitted here.

## 7. Efficiency Comparison and the Application to IoT

Refs. [14,15,16] are among the several most important batch signature schemes and improved techniques so far. Ref. [14] first introduced the idea and provide the construction based on RSA scheme. Later in [15], the authors focused on speeding up the modular exponentiation operation which is used in many DLP based signatures. In other words, the work [15] focuses on signature schemes which were built in the traditional multiplicative group, and then the authors in [16] managed to achieve the constant complexity for signature generation and verification, which does not rely on the number of messages. Our second and third schemes enjoy the same advantage as [16] regarding constant generation and verification complexity. Comparing the concrete efficiency for those schemes boils down to the issue of comparing the underlined algebraic primitives. According to [31], the key parameters, namely n,q in our lattice based scheme should be chosen at around 500 and 200,000 to achieve approximately the 128-bit level security. Generally speaking, our signature size will be larger than the ones constructed in the group where DLP or ECDLP problem is hard. According to [32], the computation of the lattice is very fast, and at the same security level the current lattice scheme will outperform the RSA and DLP or ECDLP based schemes. Most importantly, up to now, none of the previous listed batch signature schemes are able to resist against quantum attacks, which makes our scheme a very attractive choice in a long run. Lattice primitives are also being optimized by taking advantage of the modern CPU instruction such as AVX, AVX2 and so on [33], thus, the computation speed can be expected to be further improved.

In our constructions, we make use of two different approaches to integrate a group of messages to fulfill batch signatures, namely, binary tree and intersection methods. The schemes are existentially unforgeable against adaptive chosen message attacks. Table 1 shows the efficiency of our scheme regarding the different signature stages and parameter sizes in the asymptotic manner. From Table 1, it can be seen that all of the schemes require $O(n^{2}log^{3}n)$ complexity on the secret key generation, while the 2nd and 3rd constructions only take O(n) for the size of signature, which means that a batch signature is independent of the parameter *k*. For computational comparison, it can be seen that the 3rd construction requires least computational complexity, $O(n^{3})$, while others have to take *S* and other operations. In the verification stage, the 2nd and 3rd scheme are constant cost instead of being linear with *k*, unlike the 1st construction. In all aspects, 3rd construction is the most efficient, and we describe its application in wireless body sensor network, which is a typical application of IoT.

A wireless body sensor network [34] is composed of three sides: a receiver station(RS), many central control units (CCU), and many sensors (SS). A receiver station manages multiple central control units and a central control unit manages many sensors. Specifically, A patient’s body is implanted with many sensors and a central control unit, which collects human medical data and sends it to the receiver station. The receiver station is responsible for verifying data and warehousing for all central control units, that is, the receiver station checks medical data and signs all of them. When a large number of medical data, which is from different patients, come in at the same time, the receiver station will become the bottleneck of data processing. Batch signature can solve the requirement of batch signing and individual verification for patients’ medical data; the process is as follows.

**Figure 3:**
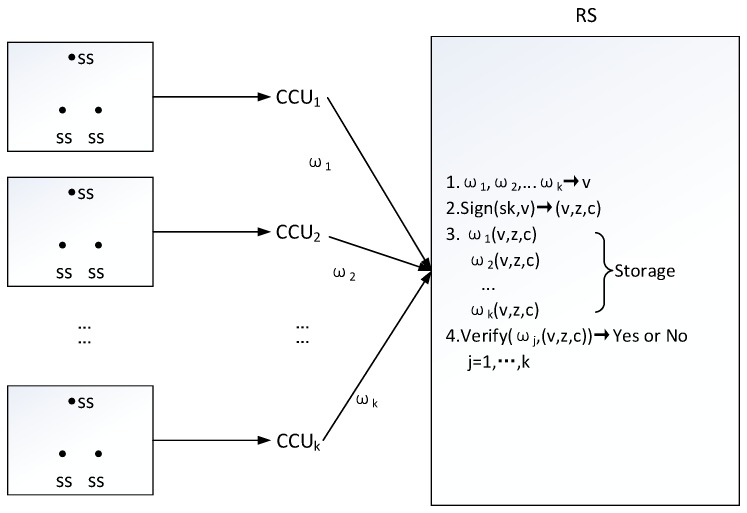
Data Flow Diagram in Wireless Body Sensor Network.

Firstly, according to the 3rd batch signature scheme, wireless body sensor network sets up system parameters and public/private keys for the receiver station. A central control unit collects medical data in real time and sends it to the receiver station. The receiver station divides *k* central control unit data into one group, such as $\left\{\varpi_{1},\cdots ,\varpi_{k}\right\}$, executes algorithm **Sign**($sk,\{\varpi_{1},\cdots ,\varpi_{k}\}$), obtain (v,z,c), store it with the message $\varpi_{i}$, i=1,⋯,k. When the *j*-th central control unit’s data $\varpi_{j}$ is called, ($\varpi_{j},\left(v,z,c\right))$ is provided, and the algorithm **Verify**($\varpi_{j},\left(v,z,c\right))$ can be invoked to verify the validity of data $\varpi_{j}$. If the answer is yes, the data is authoritative and credible. Otherwise, the data is unusable. The data flow diagram is shown in the Figure 3.

## 8. Conclusions

In this paper, we presented three new lattice-based batch signature schemes by using binary tree and intersection methods, with hash-and-sign paradigm and Fiat–Shamir transformation, respectively. Our schemes were existential unforgeability against adaptive chosen message attacks based on the difficulty of a small integer solution problem, which provided quantum security. A detailed efficiency analysis was also given, which showed that our schemes optimized the size of the public key, private key and signature. Moreover, we applied our batch signature schemes to a wireless body sensor network, which was a typical application of IoT, improved signature efficiency and security. In addition, batch signatures can also be applied to blockchain systems for speeding up the process of block signing operations, which we aim to be our next work. 

## Figures and Tables

**Table 1:** Efficiency comparison of our schemes.

Scheme (Size/Computation)	Our 1st Scheme (with Binary Tree)	Our 2nd Scheme (With Intersection Method)	Our 3rd Scheme (With FS Transformation)
sk	$O(n^{2}log^{3}n)$	$O(n^{2}log^{3}n)$	$O(n^{2}log^{3}n)$
vk	$O(n^{2}log^{2}n)$	$O(kn^{2})$	$O(kn^{2})$
signature	O(kn)	O(n)	O(n)
Sign	$\left(2k-1\right)H+S+O\left(n^{2}\right)$	$O(n^{3})+S$	$O(n^{3})$
Verify	2V+kH	2V+H	3V+H
*Anti-quantum*	*√*	*√*	*√*

Notation: H denotes Hash function, S denotes SamplePre, V denotes verification.
